# Enhancing health outcomes for Māori elders through an intergenerational cultural exchange and physical activity programme: a cross-sectional baseline study

**DOI:** 10.3389/fpubh.2023.1307685

**Published:** 2023-12-12

**Authors:** John G. Oetzel, Yingsha Zhang, Sophie Nock, Pare Meha, Huia Huriwaka, Maramena Vercoe, Tania Tahu, Joanne Urlich, Rachel Warbrick, George Brown, Shirley Keown, Poia Rewi, Bevan Erueti, Isaac Warbrick, Anne-Marie Jackson, Tracy Perry, Rangimahora Reddy, Mary Louisa Simpson, Michael P. Cameron, Brendan Hokowhitu

**Affiliations:** ^1^University of Waikato, Hamilton, New Zealand; ^2^School of Tourism Management, Sun Yat-sen University, Zhuhai, Guangdong, China; ^3^Key Laboratory of Sustainable Tourism Smart Assessment Technology, Ministry of Culture and Tourism of China, Zhuhai, Guangdong, China; ^4^Rauawaawa Kaumātua Charitable Trust, Hamilton, New Zealand; ^5^Te Runanga o Ngati Manawa, Murupara, New Zealand; ^6^Te Hiku Hauora, Kaitaia, New Zealand; ^7^Turanga Health, Gisborne, New Zealand; ^8^Te Matawai, Wellington, New Zealand; ^9^Massey University, Palmerston North, New Zealand; ^10^Auckland University of Technology, Auckland, New Zealand; ^11^University of Otago, Dunedin, New Zealand; ^12^University of Queensland, Brisbane, QLD, Australia

**Keywords:** older Māori health, indigenous aging, physical activity, health equity, health-related quality of life

## Abstract

**Background:**

The study offers baseline data for a strengths-based approach emphasizing intergenerational cultural knowledge exchange and physical activity developed through a partnership with kaumātua (Māori elders) and kaumātua service providers. The study aims to identify the baseline characteristics, along with correlates of five key outcomes.

**Methods:**

The study design is a cross-sectional survey. A total of 75 kaumātua from six providers completed two physical functioning tests and a survey that included dependent variables based in a holistic model of health: health-related quality of life (HRQOL), self-rated health, spirituality, life satisfaction, and loneliness.

**Results:**

The findings indicate that there was good reliability and moderate scores on most variables. Specific correlates included the following: (a) HRQOL: emotional support (*β* = 0.31), and frequent interaction with a co-participant (*β* = 0.25); (b) self-rated health: frequency of moderate exercise (*β* = 0.32) and sense of purpose (*β* = 0.27); (c) spirituality: sense of purpose (*β* = 0.46), not needing additional help with daily tasks (*β* = 0.28), and level of confidence with cultural practices (*β* = 0.20); (d) life satisfaction: sense of purpose (*β* = 0.57), frequency of interaction with a co-participant (*β* = −0.30), emotional support (*β* = 0.25), and quality of relationship with a co-participant (*β* = 0.16); and (e) lower loneliness: emotional support (*β* = 0.27), enjoyment interacting with a co-participant (*β* = 0.25), sense of purpose (*β* = 0.24), not needing additional help with daily tasks (*β* = 0.28), and frequency of moderate exercise (*β* = 0.18).

**Conclusion:**

This study provides the baseline scores and correlates of important social and health outcomes for the He Huarahi Tautoko (Avenue of Support) programme, a strengths-based approach for enhancing cultural connection and physical activity.

## Introduction

As the Indigenous peoples of Aotearoa New Zealand (Aotearoa), Māori comprise 17% of the population and 7% of this total are 65 and older (1). Māori face significant and stark social and health inequities relative to non-Māori populations in Aotearoa New Zealand (2, 3). For example, Māori have a life expectancy that is seven years lower than other New Zealanders and yet the major causes of mortality are preventable and treatable (4). Further, “Māori experience systematic disparities in health outcomes, determinants of health, health system responsiveness, and representation in the health sector workforce” (p. 10) (5). These health and social inequities are heightened for kaumātua (elders) who also face additional challenges including social isolation and loneliness, end-of-life concerns, and chronic health conditions (6, 7).

These health and social inequities have not decreased over the last 50 years and are largely explained by a variety of structural determinants (8). These include inequalities in social determinants (e.g., housing, income, and education), institutional discrimination from the effects of colonization, and insufficient access to health services (4, 9, 10). A key component of the explanatory factors is Te Tiriti o Waitangi (Treaty of Waitangi), which is the written agreement for the founding of Aotearoa. It is a disputed document that has different versions in English and Māori, but guaranteed Māori rights. Historically, the Treaty was not followed until 1975 with the Treaty of Waitangi Act (8). More recently, Te Tiriti has five principles: (a) Recognition and protection of tino rangatiratanga (self-determination); (b) Equity—equal access to health care and equitable outcomes; (c) Active protection—governmental protection of the first two principles; (d) Partnership—government partnering with Māori; and (e) Options—providing options for services that are grounded in te Ao Māori (Māori worldview) (2).

Today's kaumātua grew up prior to the Treaty of Waitangi Act and lived in a society that was more racist than in present and were affected by an education system that banned and punished people for embracing tikanga Māori (cultural protocols) and speaking Te Reo Māori (language) (11, 12). This colonial historical trauma contributed to health inequities through cultural dissonance or feeling of separation from their own culture (13–15). Further, kaumātua have not been recognized for their contributions to a dominant society even while Māori culture upholds elders as, “carriers of culture, anchors for families, models for lifestyle, bridges to the future, guardians of heritage, and role models for younger generations” (p. 14) (16).

The treaty principles, colonial history, and existing inequities are reasons why numerous researchers have found a need and advocate for services and programmes that are culturally appropriate and safe and that address structural determinants and structural change (7, 17, 18). However, much of the predominant narrative and specific actions to alleviate inequities are grounded in a deficit approach (19–21). The deficit approach emphasizes Indigenous communities as ‘difficulties' to be fixed relative to mainstream populations. The deficit-model approach sometimes blames Māori for the challenges they face and does not consider structural and systemic elements including loss of cultural connection resulting from colonization (8, 22, 23).

In contrast, this study offers a strength-based approach grounded in tikanga Māori and Te Ao Māori to guide solutions and this particular research. Specifically, the study focuses on kaumātua mana motuhake (actualization, autonomy, and independence) at an individual and collective level (24). He Huaraki Tautoko (Avenue of Support) is a collaboratively developed programme about intergenerational cultural knowledge exchange that also involves physical activity (24). Physical activity is correlated with various health benefits for older adults, from improved mental wellbeing (25), to a reduction in morbidity, mortality and falls (26). Particular physical activities can also be a means of strengthening cultural connection among Māori (27), while cultural activities and practices can be a driver that leads to “incidental” physical activity (28). He Huarahi Tautoko was constructed by researchers and six community providers along with their kaumātua through a participatory process. It is based on kaumātua as carriers of mātauranga (Māori knowledge systems) and involved sharing with each other along with a member of their whānau (extended family) through wānanga (learning sessions). The mātauranga included Te Reo Māori, whakapapa (genealogy), purākau (Māori lore), waiata (songs), and karakia (prayers). This cultural knowledge was selected as there is a link between cultural continuity and positive health outcomes (29–31), especially in the context of kaumātua who have a history of cultural dissonance due to colonial policies and practices. Further, this exchange of mātauranga was grounded in physical activities such as walks to significant cultural landmarks, gardening (e.g., traditional food preparation), and other cultural practices (dancing, cleaning the marae or community meeting house). Physical activity was important to enhance physical functioning and mental wellbeing. This project is a component of the Kaumātua Mana Motuhake Poi (KMMP) programme funded by the Aging Well National Science Challenge (https://www.ageingwellchallenge.co.nz/) (24).

The cultural knowledge and physical elements are important as part of a holistic model of health important for Māori communities. There are various models of Māori health that focus on a holistic perspective with perhaps the most popular being te whare tapa whā (four walls of a house) (13, 32). Te whare tapa whā was chosen by this research partnership to guide this project and includes four elements: te taha whānau, te taha hinengaro, te taha wairua, and te taha tinana (social, psychological/mental, spiritual and physical health respectively).

There are two aims of this study. The first aim is to present the baseline study from the He Huarahi Tautoko project to establish the initial comparison point and the psychometric characteristics for the measures. The second aim is to identify correlates for five outcomes related to te whare tapa whā and mana motuhake: health-related quality of life (HRQOL) and self-rated health for physical and mental wellbeing, loneliness for social health, spiritual wellbeing for spiritual health, and life satisfaction for mana motuhake. Examining the correlates of these health and wellbeing outcomes provides indicators for researchers and practitioners developing programmes and services to address health equity for kaumātua. They can also reinforce whether the He Huarahi Tautoko project is addressing key attributes.

## Methods

The larger study has a mixed methods pre-test and two post-test, staggered design; four providers receive the programme initially and two providers receive it later (24). A cross-sectional survey for the baseline measures was the study design for this specific study. The research is grounded in Kaupapa Māori (33, 34) and a participatory research approach, He Pikinga Waiora [enhancing wellbeing (35)]. Kaupapa Māori emphasizes Te Ao Māori and tikanga (36), relies on self-determination and uses mātauranga Māori and Māori epistemology (37). He Pikinga Waiora centers Kaupapa Māori, while also including a partnership model amongst researchers and communities. A partnership of six Māori social-health service providers and university researchers from four universities comprised the research team. The project is registered with the Australia New Zealand Clinical Trial Registry (ACTRN12621000541808).

### Participants

Participants were 75 kaumātua from six Māori social-health service providers across Aotearoa. We originally planned to identify a sampling frame and randomly sample participants from each provider. However, the initial planning was prior to the COVID pandemic, and we began the programme in between lockdowns for the pandemic. Kaumātua were hesitant to participate in group interactions post-lockdowns as they comprised one of the most impacted populations by the virus. Thus, the providers made the determination that they should invite all willing kaumātua to participate and it became a purposive sample. We had originally sought to recruit 15 kaumātua from each provider. Four were able to do this and two providers were only able to recruit eight and seven participants. There were 45 women and 16 men (14 did not specify) with an average age of 69.80 (SD = 7.26).

### Measures

Measures were organized around our holistic models of hauora (health) and mana motuhake. For hauora, we included the following scales: self-reported health (38, 39), HRQOL (40, 41), spirituality (42), loneliness (two items from Waldergrave et al. (43) and one item from Hayman et al. (6)), perceived and desired social support (44), relationship quality with the person participating with them in the programme (45), cultural connection (29), cultural practices (10 items created for this study), self-reported exercise hours per week (46), and physical functioning (time to complete five chair stands and time to walk 3 meters) (47, 48). We also included life satisfaction (49) and sense of purpose (50) for mana motuhake. The measures included two different types of scales: (1) 11-point semantic differential scales and (2) Likert-type scales ranging from 3–6 points. The Appendix includes the items from the survey.

There were 39 items. Participants could complete the questionnaire on their own in a paper/pencil format or have a Māori community researcher administer the survey via an interview. The survey was written with English and Māori versions (back-to-back); it was originally written in English and then translated and back-translated to ensure equivalence of Māori to English. A large font and sufficient spacing were used for ease of reading for kaumātua. Participants received a $50 voucher for completing the survey. The University of Waikato's Human Research Ethics Committee, HREC (Health) 2020#93 approved the research protocols. The data collection procedures followed a culturally appropriate approach employed in prior projects (51, 52) to provide cultural safety.

### Data analysis

Cronbach's alpha was used to assess reliability. Items from scales with low reliability were retained as individual items. Descriptive statistics included means, standard deviation and bivariate correlations. Multiple linear regression models (forward method) were employed to were run to determine the correlates of self-rated health, HRQOL, spiritual wellbeing, loneliness, and life satisfaction. The remaining items/scales were included as independent variables if they had a bivariate correlation with an outcome variable (*p* ≤ 0.10).

## Results

Descriptive statistics and Cronbach's alphas are displayed in Table 1. Spiritual wellbeing, HRQOL, and self-rated health were rescored to a 100-point response scale following prior approaches (53). Other response scores are based on the original response scale described in Table 1. The reliability for the relationship quality (α = 0.21) and social support (α = 0.37) were too low to warrant scales and thus the individual items were included for analysis.

**Table 1:** Descriptive statistics and bivariate correlations.

**Construct**	***M***	***SD***	**Self-Rated Health**	**HRQOL**	**Spiritual wellbeing**	**Life satisfaction**	**Loneliness**	**Walk time**	**Chair stands time**	**Exercise**	**Sense of purpose**	**Cultural practices**	**Cultural connection**	**Count on others daily tasks**	**Need help with daily tasks**	**Count on others emotional**	**Need more emotional support**	**Relationship quality**	**Enjoyable interaction**	**Frequent interaction**
Self-rated Health-1 item (0–100)	64.80	21.66	na																	
HRQOL-7 items (0–100)	68.74	17.21	0.64^**^	**0.87**																
Spiritual wellbeing-1 item (0–100)	78.11	18.48	0.20	0.09	**na**															
Life satisfaction-1 item (0–10)	7.88	1.79	0.26	0.17	0.31^**^	**na**														
Loneliness−3 items (1–5; 5 = low loneliness; 1 = high loneliness)	4.22	0.75	0.22	0.26^*^	0.38^**^	0.42^**^	**0.74**													
Walk time (seconds)	9.59	5.31	−0.25^*^	−0.11	−0.07	−0.05	−0.14	**na**												
Chair Stands (seconds)	14.47	7.08	−0.17	−0.19	0.03	−0.20	−0.16	0.61^**^	**na**											
Exercise-1 items (1–5)	2.97	1.14	0.32^**^	0.23^*^	0.05	−0.07	0.22	−0.25^*^	−0.05	**na**										
Sense of Purpose-3 items (1–5)	4.13	0.75	0.28^*^	0.15	0.50^**^	0.62^**^	0.36^**^	−0.16	−0.22	0.02	**0.87**									
Cultural practices-10 items (1–3)	1.90	0.53	−0.15	−0.03	0.25^*^	−0.07	−0.07	0.33^**^	0.32^**^	0.02	−0.03	**0.95**								
Cultural connection-5 items (1–5)	4.15	0.66	−0.12	0.05	0.29^*^	0.13	0.23^*^	0.20	0.08	0.00	0.30^**^	0.42^**^	0.**88**							
Count on others for help with daily tasks-1 item (1–4; 4 = always)	2.94	1.09	0.04	0.11	0.41^**^	0.33^**^	0.50^**^	0.02	−0.01	−0.03	0.18	0.25^*^	0.23^*^	**na**						
Needing more help with daily tasks-1 item (1–4; 4 = never)	3.21	0.77	0.07	−0.04	−0.05	0.17	0.08	0.02	0.01	0.14	0.27^*^	−0.18	0.01	−0.27^*^	**na**					
Count on others for emotional support-1 item (1–4; 4 = always)	3.01	1.02	0.15	0.30^*^	0.17	0.40^**^	0.50^**^	−0.06	−0.16	0.07	0.24^*^	0.02	0.11	0.63^**^	−0.16	**na**				
Needing more emotional support-1 item (1–4; 4 = never)	3.12	0.84	0.24^*^	0.12	−0.02	0.17	0.13	0.01	0.04	0.18	0.16	−0.14	−0.10	0.03	0.59^**^	−0.11	**na**			
Relationship quality with family member−1 item (1–5)	4.33	0.84	0.02	0.05	−0.01	0.22	0.01	0.19	−0.06	0.01	0.13	−0.00	0.11	−0.03	0.08	0.02	0.05	**na**		
Enjoyable interaction with family member-1 item (1–5)	4.25	0.79	−0.02	0.17	0.15	0.13	0.25^*^	−0.06	−0.03	0.08	0.01	−0.10	0.22	0.03	−0.05	−0.07	−0.08	0.47^**^	**na**	
Frequent interaction with family member-1 item (1–5)	4.05	1.32	0.19	0.24^*^	−0.13	−0.25^*^	−0.02	−0.13	−0.04	0.22	0.09	−0.05	−0.07	−0.19	0.04	−0.04	0.08	0.05	−0.12	**na**

Higher scores are high in the variable unless otherwise noted; ^*^p < 0.05; ^**^p < 0.01; Cronbach's alpha listed on the diagonal.

The correlates for the outcomes are included in Table 2. The model for self-rated health included exercise and sense of purpose as positive correlates, *F*_(2, 74)_ = 7.978, *p* = 0.001, adj *R* ^2^= 0.16. The model for HRQOL included perceived emotional support and frequent interaction with their whānau member as positive correlates, *F*_(2, 74)_ = 6.521, *p* = 0.002, adj *R*^2^ = 0.13. The model for spiritual wellbeing included sense of purpose, not needing additional help with daily tasks, and cultural practices as positive correlates, *F*_(3, 73)_ = 15.643, *p* < 0.001, adj *R* ^2^= 0.37. The regression model for life satisfaction was significant, *F*_(4, 72)_ = 23.203, *p* < 0.001, adj *R*^2^ = 0.54. Life satisfaction had a positive association with sense of purpose, perceived emotional support, and relationship quality with their whānau member; it was negatively related with frequency of interaction with their whānau member. Finally, the regression model for loneliness was significant, *F*_(5, 71)_ = 12.291, *p* < 0.001, adj *R*^2^ = 0.43. Low level of loneliness was positively associated with perceived emotional support, enjoyable interaction with their whānau member, sense of purpose, not needing additional help with daily tasks, and exercise.

**Table 2:** Multiple regression models for key health-related outcomes.

**Correlates**	***B***	***SE B***	**β**	***p***
**Self-rated health**				
Frequency of moderate to vigorous exercise	6.082	2.012	0.319	0.003
Sense of purpose	7.830	3.057	0.270	0.012
**HRQOL**				
Perceived emotional support	5.143	1.811	0.305	0.006
Frequent interaction with family member	3.270	1.396	0.251	0.022
**Spiritual wellbeing**				
Sense of purpose	11.031	2.254	0.455	0.000
Not needing more help with daily tasks	4.651	1.591	0.280	0.005
Level of proficiency using cultural practices	6.745	3.258	0.196	0.042
**Life satisfaction**				
Sense of purpose	1.352	0.194	0.566	0.000
Frequent interaction with family member	−0.410	0.106	−0.304	0.000
Emotional support	0.439	0.141	0.250	0.003
Relationship quality with family member	0.341	0.167	0.161	0.045
**Loneliness**				
Perceived emotional support	0.195	0.084	0.267	0.023
Enjoyable interaction with family member	0.231	0.083	0.246	0.007
Sense of purpose	0.244	0.089	0.244	0.008
Not needing more help with daily tasks	0.193	0.077	0.283	0.014
Frequency of moderate to vigorous exercise	0.121	0.058	0.184	0.040

## Discussion

The purpose of this study was to establish the psychometric characteristics and baseline scores for the measures in the He Huarahi Tautoko project. Descriptive statistics indicated the following: (a) high levels of life satisfaction, spiritual wellbeing, sense of purpose, and cultural connection; (b) moderate to good levels of self-rated health, HRQOL, engagement with cultural practices, and social support; and (c) low levels of loneliness and exercise frequency. There are limited direct comparisons to other populations or other studies with kaumātua in Aotearoa on these scales. The results are very similar to a recent study of kaumātua in a different project (54) illustrating why it is important to not presume deficits or using a deficit approach when working with kaumātua specifically or Māori more generally (22, 23). Further, the responses demonstrate possibilities for improvement on the scales so the He Huarahi Tautoko project can positively affect these variables.

Sense of purpose had a positive association for loneliness, life satisfaction, self-rated health, and spiritual wellbeing. Sense of purpose was operationalised as making plans, developing a sense of direction, and having goals (50). Having a sense of purpose has found to be a significant correlate for physical activity, maintaining a healthy BMI, and avoiding sleep problems in the Health and Retirement Study (USA) (55). A sense of purpose can center on a variety of factors such as caring for family, contributing to the community, or continuing to work. For kaumātua, a key aspect of sense of purpose consists of contributing to the cultural knowledge and tikanga of the community (16). Given the historical cultural dissonance experienced by kaumātua due to colonization (11, 15), the He Huarahi Tautoko project is timely and important.

Social factors such as emotional support, tangible support, and relationship quality with their family member who is participating with them in the study were key correlates for HRQOL, life satisfaction, spiritual wellbeing, and low levels of loneliness. Prior research shows social support has a positive relationship for various wellbeing and health outcomes for Indigenous and non-Indigenous populations (29, 56–60). For older people, high-quality social relationships are important for enhancing quality of life and life expectancy (61–64). The importance of social relationships was highlighted by the COVID pandemic as many kaumātua were isolated during lockdown periods (7).

Frequency of moderate to vigorous exercise was a correlate for self-rated health and low levels of loneliness. A large amount of extant literature identifies a positive association of exercise and physical activity with wellbeing, mental health, health outcomes, and cognitive functioning (65–67). The relationship with low levels of loneliness is likely due to the preference of older adults to exercise with others as a way to avoid isolation and maintain connections with others (68).

Self-rated proficiency in cultural practices was associated positively with spiritual wellbeing. Cultural practices were operationalised as knowledge and confidence in using and sharing tikanga and Te Reo Māori as well as with roles in the community. Te Ao Māori is grounded in a cultural and spiritual connection which is important for many kaumātua (16). The results of this current study are consistent with these perspectives and reinforce the aim of He Huarahi Tautoko to enhance self-rated proficiency of cultural practices. As noted earlier, the cultural dissonance experienced by kaumātua due to colonization and State policies reinforce the need for culturally resonant programmes to enhance learning about Te Reo Māori and tikanga Māori (15, 69).

There are some implications from this study for Indigenous aging and enhancing cultural practices and physical activity. This study emphasizes a holistic perspective of health including cultural, social, and spiritual elements as well as physical and mental components. Aging well for kaumātua follows Māori models of health (32) and the project this study is based on integrates these elements into the programmes for addressing cultural practices and physical activity.

Further, this study focuses on mana motuhake, which is important in the context of colonial history and not following the Treaty of Waitangi. The cultural dissonance that was created through these historical practices has negatively impacted current kaumātua (11). In addition, much of the framing around inequities that have resulted from this history is based on a deficit perspective that also has negative impacts for kaumātua (19, 69). Mana motuhake emphasizes autonomy, status, and independence of kaumātua to recognize their own concerns and thereby solutions for addressing their wellbeing. Kaumātua are acknowledged as having experience and knowledge and the keepers of Māori tikanga; thus they should be afforded the opportunity to participate in creating solutions to address health and social inequities (2).

The He Huarahi Tautoko programme was developed through a participatory process with kaumātua and kaumātua service providers that addresses key features they deem important (i.e., cultural knowledge exchange and physical activity). This programme was developed to support kaumātua mana motuhake through exchange of mātauranga with each other and with members of their own whānau. The programme addresses key aspect of health, wellbeing, physical function, and culture that are important for kaumātua. The programme is culturally grounded and culturally safe (70), which helps to ameliorate some of the negative harms created from the colonial history.

Although there are key strengths of the study and the larger project, there are a couple of limitations as well. The study uses self-reported measures which are subject to perceptual bias. However, mana motuhake suggests that kaumātua are able to describe their own wellbeing. In addition, we do include physical functioning tests to complement the self-report measures. Further, the study is a purposive sample and thus generalization to the larger population is not appropriate. There may be inherent recruitment bias as a result of the non-random participant selection.

## Conclusion

This study offered the baseline and psychometric characteristics from the He Huarahi Tautoko project, which is a programme that aims to enhance physical activity and cultural knowledge exchange for kaumātua in Aotearoa New Zealand. These results provide a baseline for later evaluation of the programme. Further, the study findings include key correlates of five wellbeing indicators grounded in the te whare tapa whā model: sense of purpose, social support and relationship quality, exercise frequency, and proficiency with Māori cultural practices. This current study illustrates key issues for kaumātua wellbeing; the He Huarahi Tautoko programme is a culturally-resonant approach that is strengths-based (rather than deficit based) to address wellbeing.

## Data availability statement

The datasets used and/or analyzed during the current study are available from the corresponding author on reasonable request.

## Ethics statement

The studies involving humans were approved by the Human Research Ethics Committee (Health), University of Waikato. The studies were conducted in accordance with the local legislation and institutional requirements. The participants provided their written informed consent to participate in this study.

## Author contributions

JO: Conceptualization, Formal analysis, Funding acquisition, Methodology, Project administration, Supervision, Writing—original draft. YZ: Conceptualization, Formal analysis, Methodology, Validation, Writing—review & editing. SN: Conceptualization, Funding acquisition, Investigation, Methodology, Supervision, Writing—review & editing. PM: Conceptualization, Funding acquisition, Investigation, Methodology, Supervision, Writing—review & editing. HH: Data curation, Investigation, Writing—review & editing. MV: Data curation, Investigation, Writing—review & editing. TT: Data curation, Investigation, Writing—review & editing. JU: Data curation, Investigation, Writing—review & editing. RW: Data curation, Investigation, Validation, Writing—review & editing. GB: Data curation, Investigation, Writing—review & editing. SK: Data curation, Investigation, Writing—review & editing. PR: Conceptualization, Methodology, Supervision, Writing—review & editing. BE: Conceptualization, Methodology, Supervision, Writing—review & editing. IW: Conceptualization, Methodology, Supervision, Writing—review & editing. A-MJ: Conceptualization, Methodology, Supervision, Writing—review & editing. TP: Conceptualization, Methodology, Supervision, Writing—review & editing. RR: Conceptualization, Funding acquisition, Methodology, Supervision, Writing—review & editing. MS: Conceptualization, Funding acquisition, Methodology, Writing—review & editing. MC: Conceptualization, Funding acquisition, Methodology, Writing—review & editing. BH: Conceptualization, Funding acquisition, Methodology, Writing—review & editing.

## Figures and Tables

**Table:** 

**Te Reo Māori**	**English approximation**
*Aotearoa*	New Zealand
*He Huarahi Tautoko*	Avenue of Support
*hauora*	Wellbeing
*He Pikinga Waiora*	Enhancing wellbeing
*karakia*	Prayers
*kaumātua*	Older people
*Kaupapa Māori*	Research/services by Māori for Māori
*mana motuhake*	Identity, autonomy, self-actualization
*Māori*	Indigenous people of Aotearoa New Zealand
*mātauranga*	Māori system of knowledge
*purākau*	Māori lore
*Te Ao Māori*	Māori worldview
*Te Reo Māori*	The Māori language
*te taha hinengaro*	Mental health
*te taha tinana*	Physical health
*te taha wairua*	Spiritual health
*te taha whānau*	Social health
*Te Tiriti o Waitangi*	Treaty of Waitangi
*te whare tapa whā*	Four walls of a house
*tikanga*	Cultural practices and protocols; cultural wellbeing
*tino rangatiratanga*	Self-determination
*waiata*	Songs
*whakapapa*	Geneology
*whānau*	Closely connected kin group/extended family
