# Supplementary material for: Enhancing health outcomes for Māori elders through an intergenerational cultural exchange and physical activity programme: a cross-sectional baseline study
Source: Front Public Health. 2023 Dec 12;11:1307685. doi: 10.3389/fpubh.2023.1307685 (PMC10749953; doi:10.3389/fpubh.2023.1307685)
Supplement: Supplementary file 1 [file Data_Sheet_1.docx]

**Appendix: Survey Items and Anchor Points in Response Scale**

**Self-rated Health** [38,39]

Overall, how would you rate your health during the past 4 weeks? (excellent, very good, good, fair, poor, very poor)

**Health-Related Quality of Life** [40,41]

During the past 4 weeks, how much did physical health problems limit your physical activities (such as walking or climbing stairs)? (not at all, very little, somewhat, quite a lot, could not do daily activities)

During the past 4 weeks, how easy was it for you to do your daily work, both at home and away from home, with your physical health? (very easy, somewhat easy, somewhat difficult, quite difficult, could not do daily work)

How much bodily pain have you had during the past 4 weeks? (none, very mild, mild, moderate, severe, very severe)

During the past 4 weeks, how much energy did you have? (very much, quite a lot, some, a little, none)

During the past 4 weeks, how much did your physical health or emotional problems limit your usual social activities with family or friends? (not at all, very little, somewhat, quite a lot, could not do social activities)

During the past 4 weeks, how much have you been bothered by emotional problems (such as feeling anxious, depressed or irritable)? (not at all, slightly, moderately, quite a lot, extremely)

During the past 4 weeks, how much did personal or emotional problems keep you from doing your usual work, school or other daily activities? (not at all, very little, somewhat, quite a lot, could not do daily activities)

**Physical Activity** [46]

In a typical week, how often do you engage in moderate to vigorous physical activity? (rarely or never, weekly but less than one hour, one to three hours per week, four to seven hours per week, more than seven hours per week)

**Spiritual Wellbeing** [42]

How would you rate your overall spiritual wellbeing? (excellent, very good, good, fair, poor, very poor)

**Loneliness**

How often do you feel lonely? (always feel lonely, mostly feel lonely, sometimes feel lonely, never feel lonely) [6]

I have really close whānau I can talk to. (strongly disagree, disagree, neutral, agree, strongly agree) [43]

I spend plenty of time with my whānau. (strongly disagree, disagree, neutral, agree, strongly agree) [43]

**Social Support** [44]

When you need extra help, can you count on whānau or others to help with daily tasks like grocery shopping, cooking, house cleaning, telephoning, giving you a ride? (always, most of the time, sometimes, never; recoded)

Thinking about the past 4 weeks, could you have used more help with daily tasks than you received? (always, most of the time, sometimes, never)

Can you count on whānau or others to provide you with emotional support? (always, most of the time, sometimes, never; recoded)

Thinking about the past 4 weeks, could you have used more emotional support than you received? (always, most of the time, sometimes, never)

**Relationship Quality** [45]

Taking things all together, on a scale from 1 to 5, where 1 is really bad and 5 is absolutely perfect, how would you describe your relationship with the person participating with you in this research?

It's easy for me to laugh and have a good time with the person participating with me in this research (strongly disagree, disagree, neutral, agree, strongly agree)

Over the last 3 months, about how often have you spent time with the person participating with you in this research doing such things as leisure activities, working on something together, or just having private talks? (not at all, less than once a month, one to three times a month, about once a week, more than once a week)

**Life Satisfaction** [49]

Please think about how you would rate your life overall with 0 = your worse possible life and 10 = to your best possible life. Where would you rate your life on this scale?

**Sense of Purpose** [50]

I have a sense of direction and purpose in life. (strongly disagree, disagree, neutral, agree, strongly agree)

I set goals for myself. (strongly disagree, disagree, neutral, agree, strongly agree)

I enjoy making plans for the future and working to make them a reality. (strongly disagree, disagree, neutral, agree,

strongly agree)

**Cultural Connection** [29]

I have a deep connection to hapū and iwi. (strongly disagree, disagree, neutral, agree, strongly agree)

I have a deep connection to the natural environment. (strongly disagree, disagree, neutral, agree, strongly agree)

I have a deep connection to our traditional cultural ways (tikanga) (strongly disagree, disagree, neutral, agree, strongly agree)

How important is your iwi to your wellbeing? (not at all important, a little important, moderately important, very important, extremely important)

I am satisfied with my cultural roles in my community. (not at all satisfied, a little satisfied, moderately satisfied, very satisfied, extremely satisfied)

**Cultural Practices** (created for this study)

(Te Kore-beginning level; Te Pō-moderate level; Te Awatea-advanced level)

My knowledge of Te Reo Māori

My knowledge of tikanga Māori

Using Te Reo Māori on a daily basis

Using tikanga Māori on a daily basis

Confidence in using Te Reo Māori on a daily basis

Confidence to use tikanga Māori on a daily basis

Telling whānau about our whakapapa in Te Reo Māori.

Telling my whānau about the whenua and significant places in Te Reo Māori

My roles in marae activities

My roles in Māori organisations
